# Psychometric properties of the acceptance and action questionnaire (AAQ II) Malay version in cancer patients

**DOI:** 10.1371/journal.pone.0212788

**Published:** 2019-02-26

**Authors:** Nurul Izzah Shari, Nor Zuraida Zainal, Ng Chong Guan, Zuraida Ahmad Sabki, Nor Aziyan Yahaya

**Affiliations:** 1 Lifestyle Science Cluster, Advanced Medical and Dental Institute, Universiti Sains Malaysia, Kepala Batas, Malaysia; 2 Department of Psychological Medicine, Faculty of Medicine, University Malaya, Kuala Lumpur, Malaysia; 3 Department of Nursing Sciences, Faculty of Medicine, University Malaya, Kuala Lumpur, Malaysia; Hong Kong Polytechnic University, HONG KONG

## Abstract

Psychological inflexibility has been found as one of the predictor to psychopathology in cancer patient. Cancer patients tend to experience psychological inflexibility as a reaction to cancer diagnosis, prognosis and treatment. Therefore, there is a need to identify psychological inflexibility due to its impact on quality of life among cancer patients.

**Objectives:**

Acceptance and action questionnaire (AAQ II) is a scale used to assess psychological inflexibility. The aim of this study is to translate AAQ II into Malay language and evaluate the psychometric properties of AAQ II Malay version.

**Methods:**

The AAQ II which has been translated into Malay language via back translation procedure was distributed to 101 cancer patients and 100 non-cancer patients. The evaluation of psychometric properties in this study included content validity index, internal consistency, parallel reliability, exploratory factor analysis, concurrent validity, sensitivity and specificity of AAQ II Malay version.

**Results:**

AAQ II Malay version has established good content validity index, acceptable internal consistency with Cronbach’s alpha value of 0.91, excellent parallel reliability and adequate concurrent validity. Exploratory factor analysis (EFA) results demonstrated AAQ II Malay version is a unidimensional factor instrument. The result of sensitivity and specificity of AAQ II Malay version indicated cancer patients who scored more than 17.5 were having significant psychological inflexibility.

**Conclusion:**

AAQ II Malay version is a reliable and valid instrument to measure psychological inflexibility among cancer patient in Malaysia.

## Introduction

Acceptance and action questionnaire (AAQ II) is an instrument to assess experiential avoidance and psychological inflexibility. Experiential avoidance can be defined as an attempt to avoid or neglect unpleasant thought, unpleasant feelings, bitter memories, uncomfortable physical sensations, and consequently lead to an action that is against one’s values and causing long-term harm [1]. Empirical evidence has found contribution of experiential avoidance on psychopathology in cancer patients [2,3]. Cancer patients commonly experienced experiential avoidance as a reaction to cancer diagnosis, prognosis and treatment [4]. Experiential avoidance is found to be one of the coping strategies among cancer patients and become a predictor to psychosocial problem such as depression, anxiety and distress [5,6]. The attempts to avoid the unpleasant thought, feeling and memories related to cancer elevate their discomfort and lead to the lack of involvement in their valued activities and life. As a result, this circumstance becomes the source of psychosocial and emotional issues such as distress, anxiety and depression [7]. Hence, it affects their quality of life [2,3], perceived health, psychosocial life [2] and career [8].

Meanwhile, psychological inflexibility refers to rigid psychological reaction against one's value in order to avoid distress, uncomfortable feeling and thought and tend to ignore the present moment [9]. Psychological inflexibility has been suggested as an important element in the etiology and the preservation of psychopathology [10,11]. Psychological inflexibility has positive correlation with symptoms burden that commonly experienced by cancer patient including pain, fatigue, sleep disturbance, cognitive concerns, anxiety, and depressive symptoms. The increase of psychological inflexibility among cancer patient will heighten their symptom burden and leads to the lack of engagement with valuable activities and relationship [12]. In contrast, psychological flexibility is the ability to fully experience present moment that includes one’s thoughts and feelings without struggling to control or change it, and the ability to either persist or change behavior in the given context that is consistent with one’s values and goals [10]. Individual who is psychologically flexible able to disengage with unworkable thought and actions, enjoy the present moment, accept the thing and circumstance that beyond control, and choose to live in held values and move closely to those values [13]. Psychological flexibility is important element in cancer survivorship particularly in prevent enhancement of psychological problems such as anxiety, depression and negative affect [14].

Previous studies demonstrated that AAQ II is the most widely used instrument to measure the effectiveness of Acceptance and Commitment Therapy (ACT) due to its high reliability, validity and sensitivity to identify changes in experiential avoidance [15–17]. ACT is a psychological intervention that specifically developed to manage experiential avoidance and encourage psychological flexibility.

### Measurement of experiential avoidance

AAQ II is a revised version of original version of AAQ due to its limitations. The original version of AAQ consists of 16 items and 9 items (two versions). The usage of AAQ was proven useful to measure psychological inflexibility and to predict the quality of life with average effect size in the variety of psychopathology illnesses such as depression, anxiety, work performance, pain and etc. However, the original version of AAQ encountered two main weaknesses which included low to moderate internal consistency and unstable factor structure [9]. The original version has been revised and the stable factor with high internal consistency across variety of population namely AAQ II [9]. AAQ II has been acknowledged with its high internal consistency and reliability across different types of psychopathology illnesses and different target groups. Unlike AAQ, AAQ II consisted of 7 items and a stable unidimensional measure across different population and time. The unidimensional factor indicates that AAQ II is a specific measurement tool for experiential avoidance. The AAQ II also has been translated multiple languages namely Turkish [15], Chinese [16], French, Italian, Arabic and etc. There were few studies that examined the differences between clinical and nonclinical population [15,17,18]. There were inconsistent results of the scores’ differences between clinical and nonclinical sample. However most of the studies demonstrated significantly higher scores in clinical sample compared to non-clinical sample [15,18–20]. However there were few limitations of these studies that need to be considered such as these studies were using heterogeneous sample for clinical sample.

### Overview of the present study

Even though AAQ II has been translated into multiple languages, but to the best of our knowledge, there is still no AAQ II Malay version that has been validated. Therefore, the overall aim of this study is to validate the AAQ II Malay version. The finding will provide evidence of the robustness of AAQ II and the generality of construct experiential avoidance in Asian population particularly in Malaysia which is a multi-racial and multicultural country. The first aim of this study is to examine the content validity index (CVI) of AAQ II Malay version. Content validity index (CVI) [21] is calculated to determine the content validity of the AAQ II Malay version. Content validity measured the comprehensiveness and representativeness of the content domain of an instrument [22]. Content validity is important because it is concerning the degree to which the content of the construct is adequately represented by the items in the instrument [23]. In the present study, content validity was conducted to obtain the agreement among subject matter experts for each item (I-CVI) in the instrument and for the whole instrument (S-CVI).

Then, the second aim is to explore internal consistency, to examine the parallel form reliability and to explore the potential factors in AAQ II Malay version. The parallel form reliability was measured by assessing the correlation between AAQ II English version and AAQ II Malay version. Factor analysis was conducted via exploratory factor analysis (EFA) using sample of cancer patients. The third aim is to explore the concurrent validity of AAQ II Malay version. The concurrent validity was measured by assessing the relationship of AAQ II Malay version with other instruments; The Hospital Anxiety and Depression Scale for Anxiety (HADS-A), The Hospital Anxiety and Depression Scale for Depression (HADS-D) and White Bear Suppression Inventory (WBSI). The fourth aim of this study is to examine the sensitivity and specificity of AAQ II Malay version in cancer patients by comparing it with AAQ II in non-cancer patients.

## Methodology

### Research design

In measuring content validity index, descriptive research design with purposive sampling method was used to select subject matter experts to evaluate content validity. In measuring reliability, validity, sensitivity and specificity, descriptive research design with random sampling method were used to select respondents.

### Sample

101 cancer patients were recruited from the oncology department in University Malaya Medical centre (UMMC) and National cancer institute (NCI). The inclusive criteria for this validation study are cancer patient in outpatient department (newly diagnosed and recurrent), above 18 years old, bilingual (Malay and English), and no prior psychiatric history. Meanwhile, 100 non cancer respondents were randomly selected at public places like in community hall and hospital.

### Procedure

The study was conducted starting from October 2016 to December 2017 at oncology clinic UMMC and a daycare at NCI. The ethical approval was obtained from Medical Ethic Committee (MEC) of the University Malaya Medical Centre and National Medical Research Register (NMRR).

Respondents who met inclusion criteria were approached and explained on regards to research procedure. Once the written informed consent was obtained, a self-administered questionnaire was distributed.

#### Back translation procedure

AAQ II was translated into Malay Language via back translation. By translating to respondents’ native languages, it will facilitate respondents in understanding the questionnaire. The English version of AAQ-II was translated into Malay version by 2 independent translators (first and second translators). First and second translators are experts in languages (Malay and English). Then, the third translator who is expert in language and psychology evaluated the two versions of Malay translation and chose the most accurate interpretation of the real meaning of the translation in the target language. Then, another two independent translators (fourth and fifth translators) translated back from Malay version (determined by third translator) to English version. The fourth and fifth translators have no prior knowledge of the original version. The translated versions were compared with the original English version to evaluate the equivalence of meaning between the original content and translated version.

#### Expert review

Once translated, AAQ II Malay version was submitted to six subject matter experts in psychology and oncology; four clinical psychologists, one psychiatrist (psycho-oncology) and one oncology nurse. The experts evaluated the items in the AAQ II based on clarity and relevance on 4 points Likert scale. The experts reviewed the questionnaire and commented the questionnaire based on the relevancy of the item to represent the measured construct, accuracy and clarity of the translation. They identified the item that did not clearly express the accurate meaning or did not fit with Malay idiomatic expressions and gave suggestions for improvement. In term of clarity, the items were rated based on 1) not clear, 2) item need some revision, 3) clear but need minor revision, or 4) very clear. In term of relevancy, the items were rated based on how adequately the items matched the main construct of AAQ II Malay version by using the following four-point scale: (1) not relevant, (2) item need some revision (3) relevant but need minor revision, or (4) very relevant.

### Questionnaires

#### Socio-demographic

Respondents were required to provide their socio-demographic information such as gender, age, employment status, educational background, diagnosis and stage of cancer.

#### Malay version of Acceptance and action II

7 items AAQ II Malay version as outlined in the final Malay version was used. The items as shown in S1 Appendix were rated on a 7 point Likert type scale from 1 (never true) to 7 (always true). Higher score on AAQ II indicates a greater level of experiential avoidance.

#### Acceptance and action II (English version)

AAQ II (S2 Appendix) was originally from AAQ developed by Hayes et al, [24] and has been revised by Bond et al. [9]. AAQ II is a unidimensional scale with 7 items and is rated based on 7 point Likert scale. AAQ II has good internal consistency (α = 0.88) [9] and good test retest reliability over 3 and 12 months at 0.81 and 0.79 respectively. The translated version of AAQ II demonstrated acceptable reliability in Chinese version with α = 0.86 [16], Turkish version with α = 0.84[15] and Greek version with α = 0.92[17].

#### White Bear Suppression Inventory (WBSI)

White Bear Suppression Inventory is developed by Wegner & Zanakos [25] in 1994. WBSI measures the tendency to suppress the undesirable thought including obsessive thinking and negative affect related with depression and anxiety. WBSI consists of 15-items and rated based on 5-point Likert scale. Higher scores on the WBSI scale indicate a higher tendency to suppress thoughts. The WBSI scores demonstrated a good internal consistency with α > 0.87 [25].

#### Hospital anxiety depression scale malay version (HADS-M)

The Hospital Anxiety and Depression Scale is developed by Zigmond and Snaith [26] in 1983. HADS measures mood disorder particularly depression and anxiety. HADS consists of 14 items; 7 items for anxiety scale (HADS-A) and 7 items for depression scale (HADS-D). Each item is evaluated on a 4 point (0–3) Likert scale and a higher score indicates higher symptoms of anxiety and depression. HADS Malay version has a good internal consistency with α = 0.82 [27].

#### Statistical analysis

The proportion is used to analyse content validity index of AAQ II particularly to calculate content validity index item level (I-CVI), content validity index scale-level (S-CVI) and modified Kappa statistic. Item level CVI (I-CVI) for relevancy and clarity of each item were calculated by dividing the number of experts who evaluate the item as 3 or 4 with the number of content experts. If the score of I-CVI is higher than 0.79, the item is considered as excellent and appropriate, score between 0.7 and 0.7 indicates that the item needs a revision and score below than 0.7 indicates that the item should be eliminated [28,29]. In order to calculate the S-CVI, the sum of I-CVIs was divided by the total number of items. The score of S-CVI which is 0.9 and higher is considered as appropriate and excellent [23,30]. Modified kappa statistic (k*) is defined as an index of agreement among the subject matter experts that the item is relevant and clear [30]. In term of calculation for modified Kappa statistic, the probability of chance agreement (pc) will be calculated for each item by the following formula:

$$\text{Pc}=\left[\text{N/A}\left(\text{N-A}\right)\right]*0{\text{.5}}^{\text{N}}$$

In this formula, N = number of experts and A = number of experts who agree that the item is relevant. After that, Kappa will be computed by entering the values of probability of chance agreement (Pc) and CVI of each item (I-CVI) by following formula:

K=(I-CVI-Pc)/(1-Pc).

The interpretation of Kappa value above 0.74 is considered as excellent, between 0.60 and 0.74 is considered as good, and the value between 0.40 and 0.59 is considered as fair [31].

**Fig 1:**
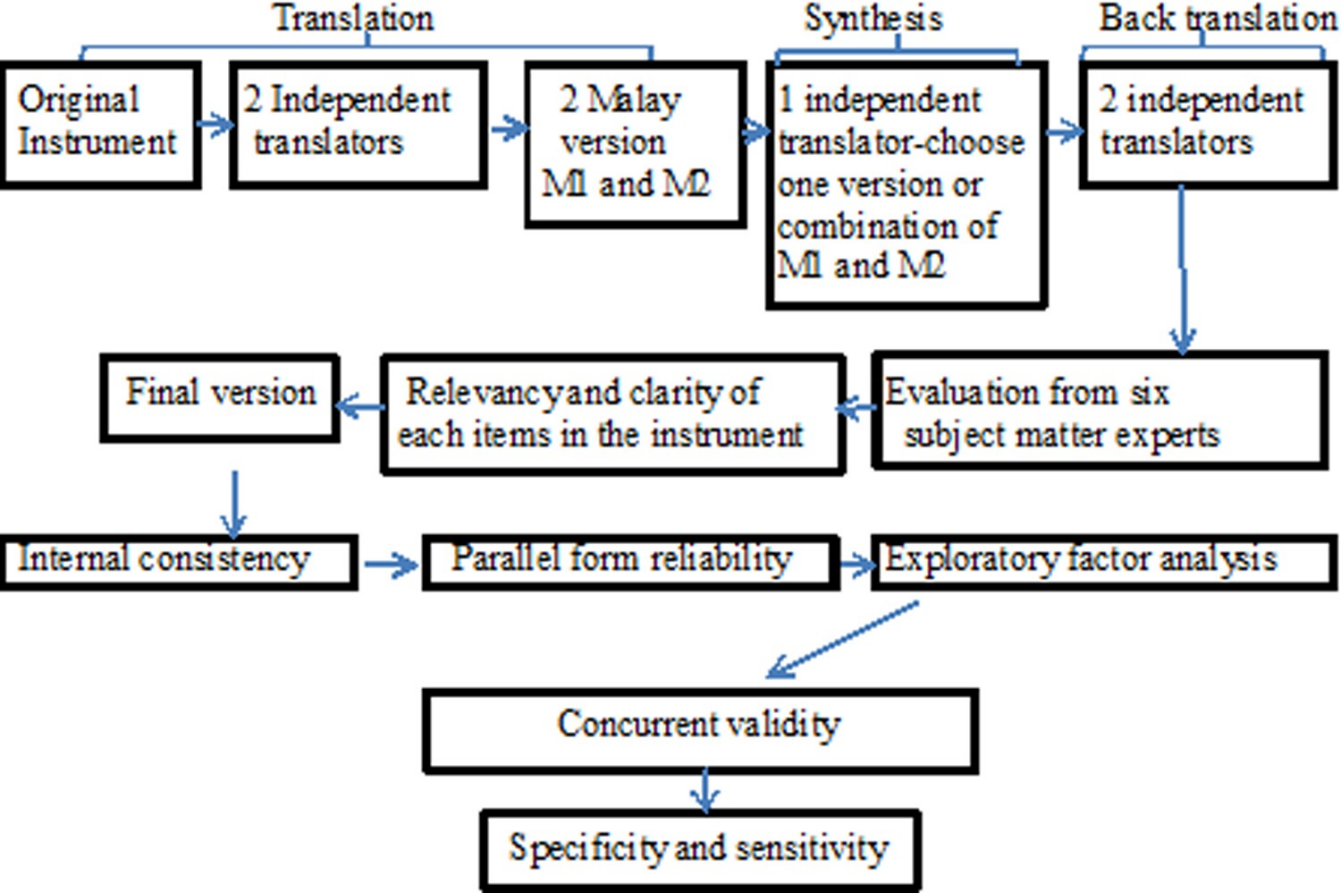
Flow of Psychometric properties study.

Statistical Package for Social Sciences version 22.0 (SPSS) was used to analyse reliability, validity, sensitivity and specificity. Data pertaining socio-demographic were computed using descriptive statistic. In the evaluation of internal consistency of AAQ II Malay version, Cronbach’s alpha and homogeneity of the scales were assessed. The intra-class correlation coefficient (ICC) was computed to examine the parallel form reliability between AAQ II and AAQ II Malay version. Principles axis factoring was performed to extract the dimensionality of AAQ II Malay version. In establishing concurrent validity, the Spearman’s test was performed to examine the correlation between AAQ II Malay version with HADS-A, HADS-D, and WBSI. Analysis of covariance was performed to examine the differences between cancer patients and non-cancer respondents with control of gender, age and educational level. The rates of sensitivity and specificity were analyzed via area under the curve (AUC) and the Youden Index. Cut-off point of AAQ II Malay version for cancer patients was determined based on the highest score of Youden Index (J). The flow of study was as shown by (Fig 1).

## Result

Table 1 shows demographic information across respondents with cancer and without cancer. 101 respondents with cancer (14.9% male and 85.1% female) and 100 respondents without cancer (31% male and 69% female) were recruited. Mean age for cancer respondents is 48.32 and mean age for non-cancer respondents is 43.44. Only age has significant mean difference with total scores of AAQ II Malay version.

**Table 1:** Demographic information.

Variables	Cancer		Non cancer	
	Frequency	Percentage	Frequency	Percentage
Age (mean±SD)	48.32±10.69		43.44±13.18	
Gender				
Male	15	14.9	31	31
Female	86	85.1	69	69
Education level				
Primary	8	7.9	14	14
Secondary	44	43.6	39	39
Tertiary	49	48.5	47	47
Employment Status				
Employed	55	54.5	58	58
Unemployed	46	45.5	42	42
Diagnosis				
Reproductive cancers	78	77.2		
Respiratory cancers	10	9.9		
Gastrointestinal cancers	8	7.9		
Bone cancer	1	1		
Brain tumor	1	1		
Thyroid cancer	1	1		
Bladder cancer	1	1		
Liver cancer	1	1		
Stage				
Stage 1	5	5.0		
Stage 2	33	32.7		
Stage 3	42	41.6		
Stage 4	21	20.8		

Reproductive cancer including breast, ovarian and endometrium cancer; Respiratory cancers including lung and nasopharyngeal cancer; Gastrointestinal cancers including colon, rectal and esophagus cancer.

### Content validity

Content validity index (CVI) was calculated via agreement of six subject matter experts for each item (I-CVI) in the instrument and for the whole instrument (S-CVI). Content validity index item level (I-CVI) scores for all items in respect of relevance, five items had perfect scores and two items were 83% relevance for experiential avoidance construct. In term of clarity, six items scored perfect score and only one item scored 0.83. After determining the CVI-I, modified kappa statistic were calculated, 5 items had perfect score (1) for k*index for relevance and two items scored 0.82 for relevance. Meanwhile, six items had perfect score for k*index and one item scored 0.82 for clarity. Then, the score for entire items in the instrument was calculated after obtaining the I-CVI for each item (Table 2). The content validity index scale level (S-CVI) for relevance and clarity were 0.95 and 0.97 respectively.

**Table 2:** Content validation of AAQ II Malay version.

Item	Relavence		Clarity		Results
	I-CVI	Modified kappa statistic	I-CVI	Modified kappa statistic	
1	1	1	1	1	Validated
2	1	1	1	1	Validated
3	0.83	0.82	0.83	0.82	Validated
4	1	1	1	1	Validated
5	0.83	0.82	1	1	Validated
6	1	1	1	1	Validated
7	1	1	1	1	Validated
	S-CVI	0.95	S-CVI	0.97	

I-CVI, Item level content validity index; S-CVI, Scale level content validity index

### Reliability

The AAQ II Malay version provided good internal consistency which Cronbach’s alpha value was 0.91 for cancer respondents and 0.90 for non-cancer respondents. The mean values were around 3.00 indicating that overall there was a moderate level of psychological inflexibility. The highest correlation for each item with at least one other item in the construct is between 0.3 and 0.9. Hence, the items correlate adequately in the construct. The lowest corrected item-total correlation (CITC) value was 0.605> 0.3. Elimination of any item in the AAQ II Malay version scale would not increase the Cronbach’s alpha value. Intra-class correlation coefficient ICC between AAQ II (English) and AAQ II Malay version (ICC = 0.938, p<0.00) indicated excellent parallel reliability.

### Dimensionality of the AAQ II Malay version

Values of the Kaiser-Meyer-Olkin which were more than 0.5 (0.874) indicated the sampling were adequate and a significant value (*p* < .001) of Barlett’s Test of Sphericity also suggested that relationship exist between at least some of the subscales and the data were suitable for factor analysis. A single factor was extracted using principle axis factoring and that explained 59.78% of the variation in the 7 items (eigen value > 1.00). The minimum factor loading was 0.640>0.5.

### Concurrent validity

The AAQ II Malay version was significantly correlated with the HADS-D (p<0.01), HADS-A (p<0.01) and WBSI (p<0.01) scores (Table 3). Hence, concurrent validity of AAQ II Malay version was established.

**Table 3:** Spearman's rho correlations (r) among the AAQ II Malay version, HADS-A and HADS-D.

Instruments	AAQ II-M	HADS-D	HADS-A	WBSI
AAAQ II Malay version	1.000	0.649**	0.643**	0.474**
HADS-D	0.649**	1.000	0.757**	0.498**
HADS-A	0.643**	0.757**	1.000	0.517**
WBSI	0.474**	0.498**	0.517**	1.00

AAQ II-M, Acceptance and action questionnaire II Malay version; HADS-D, The Hospital Anxiety and Depression Scale- Depression; HADS-A, The Hospital Anxiety and Depression Scale- Anxiety; WBSI, White Bear Suppression Inventory.

**Correlation is significant at the 0.01 level (2-tailed)

There was a significant difference in mean in the AAQ II Malay version scores [F(1,199) = 51.34, p = 0.000] between the cancer and non-cancer respondents when the age was adjusted. Cancer patients (M = 20.63, SD = 8.03) reported significantly higher AAQ II Malay version scores than non-cancer respondents (M = 14.17, SD = 4.90).

### Specificity, positive predictive value, and negative predictive value of AAQ II Malay version

Based on ROC curve analysis (Table 4), the area under the curve was 0.747 (95% CI = 0.68–0.82). Based on the Youden Index, the optimal cut off score to distinguish respondents with cancer and without cancer was >17.5 with a sensitivity of 63% and specificity of 78% (Table 5).

**Table 4:** Area under the curve.

Area	Std. Error^a^	Asymptotic Sig.^b^	Asymptotic 95% Confidence Interval	
			Lower Bound	Upper Bound
0.747	0.035	0.000	0.677	0.816

a. Under the nonparametric assumption

b. Null hypothesis: true area = 0.5

**Table 5:** Coordinates of the receiver operating characteristic (ROC) curve.

Positive if Greater Than or Equal Toa	Sensitivity	1—Specificity	Specificity	Youden Index
6	1	1	0	0
7.5	0.95	0.9	0.1	0.05
8.5	0.921	0.87	0.13	0.051
9.5	0.901	0.82	0.18	0.081
10.5	0.861	0.8	0.2	0.061
11.5	0.851	0.71	0.29	0.141
12.5	0.842	0.66	0.34	0.182
13.5	0.802	0.49	0.51	0.312
14.5	0.762	0.36	0.64	0.402
15.5	0.713	0.3	0.7	0.413
16.5	0.673	0.26	0.74	0.413
**17.5**	**0.634**	**0.22**	**0.78**	**0.414**
18.5	0.545	0.17	0.83	0.375
19.5	0.535	0.14	0.86	0.395
20.5	0.475	0.11	0.89	0.365
21.5	0.436	0.11	0.89	0.326
22.5	0.406	0.11	0.89	0.296
23.5	0.386	0.06	0.94	0.326
24.5	0.337	0.03	0.97	0.307
25.5	0.287	0.03	0.97	0.257

The best cut-off point was considered that at which the closest number of J = +1 was obtained in the Youden Index (J)

## Discussion

The purposes of this study were to translate, adapt and validate the AAQ II Malay version. The first objective was to evaluate the content validity index of AAQ II Malay version. Content validity implied the robustness of the interpretation of the items in the instrument to reflect and represent the construct. In this context, the results of this current study were determined by CVI and modified Kappa coefficient. The findings reveal that all items scored I-CVI equal to 0.83 and above. According to Polit & Beck [29], the I-CVI scores that fall above 0.79 are considered as excellent and appropriate. The S-CVI for both relevance and clarity that exceed 0.9 indicate as appropriate and excellent. The results from modified kappa statistic demonstrated the index of agreement between subject matter experts in AAQ II items that were relevant and cleared, were 82% and reached excellent level. Based on the results, all items in the instrument AAQ II were validated.

The findings in this study revealed that AAQ II Malay version has good internal consistency with Cronbach’s alpha value of 0.91 (cancer respondents) and Cronbach’s alpha value of 0.90 (non-cancer respondents). This result is comparable with the original AAQ II with α = 0.84 [9], AAQ II Turkish version with α = 0.84 [15], AAQ II Chinese version with α = 0.82 [16], AAQ II Portuguese version with α = 0.89 [20] and AAQ II Greek version with α = 0.92 [17]. The high internal consistency of AAQ II Malay version among cancer and non-cancer respondents indicated that the items in this scale measured the same construct namely experiential avoidance. In addition, the result of parallel for reliability also demonstrated excellent reliability between Malay version and original version of AAQ II. This result supports that AAQ II Malay version is acutely assessed and the construct of experiential avoidance is in line with the original version.

Exploratory factor analysis (EFA) was conducted to determine the dimensionality of AAQ II Malay version. The results of this study demonstrated that there is a single factor that accounted the large proportion of variance (59.78%) in AAQ II Malay version. The result of unidimensional factor is similar with the study conducted by Bond et al., [9], Karekla & Michaelides [17] and Zhang et al., [16].

In term of examining the concurrent validity, the correlation between AAQ II Malay version and HADS-A, HADS-D and WBSI were performed. The results demonstrated the moderate positive correlations between experiential avoidance and anxiety, depression and suppression thoughts related to anxiety and depression. These correlations supported the evidence that experiential avoidance as significant element in the etiology and the preservation of psychopathology problems such as depression and anxiety.

In this study, the mean total score of AAQ II Malay version was reported as significantly higher than non-cancer respondents. However the mean of clinical sample in this study was lower than clinical sample in the study conducted by Bond et al., [9] (M = 28.34) and other studies conducted in Portuguese population (M = 24.72) [18], Turkish population (M = 26.17) [15]. The novelty finding of this study is that the results provide information on the sensitivity and the specificity of AAQ II Malay version among cancer patients. To the best our knowledge, there is still no study that examines the cut off score of AAQ II for cancer patients. Though AAQ II is not designed as a diagnostic tool, but the cut off score can be used as a reference to identify the scores of AAQ II Malay version that is associated with the significant indicator of experiential avoidance among cancer patients. The result indicated that the score of AAQ II Malay version was more than 17.5, the cut off point for cancer patients that experienced significant experiential avoidance. The cut off score of AAQ II Malay version for cancer patients was lower than the cut off score of AAQ II for sample that was seeking treatment for substance misuse which fall between 24 to 28 [9]. In spite of different type of clinical sample, the lower mean total score and cut off score may be related with cultural differences between Malaysian and others countries in term of how Malaysian expressed their feeling and thought especially the negative thought and feelings. Malaysians, regardless the races, tend to be very careful when exhibiting their negative emotions especially when they are emotionally depressed [32]. They tend to remain silent as well as underrated their emotion and feeling due to culturally acquired rules of interaction [32].

There are few limitations in this study. First, this is a cross sectional study, therefore it is impossible to identify the causal factors and effect of experiential avoidance in cancer patients. Second, the cancer patients in this study were recruited from oncology clinic and day-care using convenience sampling, thus generalization of the findings is one of the possible limitations. Lastly, small sample size could explain the moderate factor loading (0.64) in the item six in the instrument found in this study. Larger sample sizes in future possibly will yield higher factor loading for this item.

## Conclusion

In spite of these limitations, the AAQ II Malay version demonstrated acceptable and promising psychometric properties of AAQ II Malay version. The results in term of dimensionality, reliability and validity were consistent with previous studies [9,15,16]. In addition, the current study also provided the empirical evidence on sensitivity and specificity of AAQ II Malay version in cancer patients that is devoted to Malaysian population.

## Supporting information

S1 Appendix Soal selidik penerimaan dan tindakan II versi Bahasa Melayu (AAQ II). (PDF)

S2 Appendix Acceptance and action questionnaire II (AAQ-II). (PDF)
