# Supplementary material for: Psychometric properties of the acceptance and action questionnaire (AAQ II) Malay version in cancer patients
Source: PLoS One. 2019 Feb 26;14(2):e0212788. doi: 10.1371/journal.pone.0212788 (PMC6391017; doi:10.1371/journal.pone.0212788)
Supplement: S1 Appendix — (PDF) [file pone.0212788.s001.pdf]

S1 Appendix. Soal selidik penerimaan dan tindakan (AAQ II)

Berikut adalah senarai beberapa penyataan. Sila tentukan sejauh mana setiap penyataan benar untuk kamu dengan membulatkan nombor disebelahnya. Gunakan skala berikut untuk buat pilihan.

Bil	Perkara	Tidak benar	Sangat jarang	Jarang	Kadang -kadang	Selalunya benar	Hampir sentiasa benar	Sentiasa benar
1	Pengalaman dan kenangan yang menyakitkan menyukarkan saya untuk menjalani kehidupan yang saya hargai							
2	Saya takut dengan perasaan saya							
3	Saya bimbang tidak dapat mengawal kebimbangan dan perasaan saya							
4	Kenangan lalu yang pahit menghalang saya daripada mempunyai kehidupan yang memuaskan.							
5	Emosi menimbulkan masalah dalam hidup saya							
6	Kebanyakan orang seolah-olah mampu mengendalikan hidup mereka lebih baik daripada saya							
7	Kebimbangan merupakan halangan bagi saya untuk berjaya.							
