# Supplementary material for: Psychometric properties of the acceptance and action questionnaire (AAQ II) Malay version in cancer patients
Source: PLoS One. 2019 Feb 26;14(2):e0212788. doi: 10.1371/journal.pone.0212788 (PMC6391017; doi:10.1371/journal.pone.0212788)
Supplement: S2 Appendix — (PDF) [file pone.0212788.s002.pdf]

S2 Appendix. Acceptance and action questionnaire II (AAQ-II)

Below you will find a list of statements. Please rate how true each statement is for you by ticking the appropriate box beside each item. Use the scale below to make your choice.

No	Item	Never true	Very seldom true	Seldom true	Sometimes true	Frequently true	Almost always true	Always true
1	My painful experiences and memories make it difficult for me to live a life that I would value.							
2	I'm afraid of my feelings.							
3	I worry about not being able to control my worries and feelings.							
4	My painful memories prevent me from having a fulfilling life.							
5	Emotions cause problems in my life							
6	It seems like most people are handling their lives better than I am.							
7	Worries get in the way of my success.							

(Bond et al, 2011)
